# Hypoxia-inducible factor 1alpha and vascular endothelial growth factor in Glioblastoma Multiforme: a systematic review going beyond pathologic implications

**DOI:** 10.32604/or.2024.052130

**Published:** 2024-07-17

**Authors:** DIMITRA P. VAGELI, PANAGIOTIS G. DOUKAS, KERASIA GOUPOU, ANTONIOS D. BENOS, KYRIAKI ASTARA, KONSTANTINA ZACHAROULI, SOTIRIS SOTIRIOU, MARIA IOANNOU

**Affiliations:** 1 Department of Surgery, Yale University, New Haven, CT 06510, USA; 2 Department of Pathology, Faculty of Medicine, School of Health Sciences, University of Thessaly, Larissa, 41500, Greece; 3 Department of Medicine, Rutgers/Saint Peter’s University Hospital, New Brunswick, NJ 08901, USA; 4 Department of Neurology, Army Share Fund Hospital (NIMTS), Athens, 11521, Greece; 5 Laboratory of Embryology, Faculty of Medicine, School of Health Sciences, University of Thessaly, Larissa, 41500, Greece

**Keywords:** Glioblastoma multiforme (GBM), Astrocytoma Grade III, Astrocytoma Grade IV, Hypoxia-inducible factor 1alpha (HIF-1α), Vascular endothelial growth factor (VEGF)

## Abstract

Glioblastoma multiforme (GBM) is an aggressive primary brain tumor characterized by extensive heterogeneity and vascular proliferation. Hypoxic conditions in the tissue microenvironment are considered a pivotal player leading tumor progression. Specifically, hypoxia is known to activate inducible factors, such as hypoxia-inducible factor 1alpha (HIF-1α), which in turn can stimulate tumor neo-angiogenesis through activation of various downward mediators, such as the vascular endothelial growth factor (VEGF). Here, we aimed to explore the role of HIF-1α/VEGF immunophenotypes alone and in combination with other prognostic markers or clinical and image analysis data, as potential biomarkers of GBM prognosis and treatment efficacy. We performed a systematic review (Medline/Embase, and Pubmed database search was completed by 16th of April 2024 by two independent teams; PRISMA 2020). We evaluated methods of immunoassays, cell viability, or animal or patient survival methods of the retrieved studies to assess unbiased data. We used inclusion criteria, such as the evaluation of GBM prognosis based on HIF-1α/VEGF expression, other biomarkers or clinical and imaging manifestations in GBM related to HIF-1α/VEGF expression, application of immunoassays for protein expression, and evaluation of the effectiveness of GBM therapeutic strategies based on HIF-1α/VEGF expression. We used exclusion criteria, such as data not reporting both HIF-1α and VEGF or prognosis. We included 50 studies investigating in total 1319 GBM human specimens, 18 different cell lines or GBM-derived stem cells, and 6 different animal models, to identify the association of HIF-1α/VEGF immunophenotypes, and with other prognostic factors, clinical and macroscopic data in GBM prognosis and therapeutic approaches. We found that increased HIF-1α/VEGF expression in GBM correlates with oncogenic factors, such as miR-210-3p, Oct4, AKT, COX-2, PDGF-C, PLDO3, M2 polarization, or ALK, leading to unfavorable survival. Reduced HIF-1α/VEGF expression correlates with FIH-1, ADNP, or STAT1 upregulation, as well as with clinical manifestations, like epileptogenicity, and a favorable prognosis of GBM. Based on our data, HIF-1α or VEGF immunophenotypes may be a useful tool to clarify MRI-PET imaging data distinguishing between GBM tumor progression and pseudoprogression. Finally, HIF-1α/VEGF immunophenotypes can reflect GBM treatment efficacy, including combined first-line treatment with histone deacetylase inhibitors, thimerosal, or an active metabolite of irinotecan, as well as STAT3 inhibitors alone, and resulting in a favorable tumor prognosis and patient survival. These data were supported by a combination of variable methods used to evaluate HIF-1α/VEGF immunophenotypes. Data limitations may include the use of less sensitive detection methods in some cases. Overall, our data support HIF-1α/VEGF’s role as biomarkers of GBM prognosis and treatment efficacy.

## Introduction

Glioblastoma multiforme (GBM) is considered the most common, malignant, primary brain tumor, which tends to relapse in some patients, even after an aggressive combination of therapies due to the molecular heterogeneity of the disease [1–3]. GBM tumors are characterized by a hypoxic microenvironment [4]. In the tumor microenvironment of GBM, hypoxia-inducible factors (HIFs) expression is elevated promoting angiogenesis, which plays a critical role in GBM aggressiveness and poor prognosis [5]. Drug resistance is also a GBM characteristic, due to low drug delivery to the tumor. However, the mechanisms behind treatment failure have been partly elucidated, with hypoxia playing the most crucial role, by promoting both chemo- and radio-resistance [6]. Therefore, although there are several signaling pathways implicated in the development of GBM, the role of the hypoxia-inducible factor-1alpha (HIF-1α)/vascular endothelial growth factor (VEGF) pathway is important in GBM progression and therapeutic strategies.

This systematic review article outlines the current evidence on the role of HIF-1α and VEGF alone and in combination with other prognostic markers or clinical and image analysis data, as potential biomarkers of GBM prognosis and treatment efficacy. Histopathological evidence of HIF-1α and VEGF in tumor cells is presented along with the possible involvement in tumor prognosis, alongside cellular mechanisms, clinical and imaging analysis data, and therapeutic approaches. In addition, we demonstrate the current knowledge regarding the association between HIF-1α/VEGF expression in GBM specimens and the molecular subtype of the tumor. Insights into the role of HIF-1α and VEGF immunophenotypes in GBM could support their use as biomarkers in GBM treatment efficacy.

GBM is a grade IV glioma brain tumor, and it is the most common type of glioma derived from neural stem cells (NSC), NSC-derived astrocytes, and oligodendrocyte precursor cells (OPC) [2,3]. GBM is characterized as an incurable cancer type with a 5-year survival of only 7.2% [1], even though the diagnosis is followed by an aggressive combination of therapies, like surgical resection, adjuvant radiation therapy (RT) with concurrent and adjuvant temozolomide (TMZ) treatment [7,8]. Even after drastic treatment, GBM can relapse due to the molecular heterogeneity of the disease [9] and its ability to microscopically infiltrate the surrounding and distant healthy tissue, making gross total resection challenging [7]. GBM is divided into two distinct types: primary GBM and secondary GBM [9].

The histopathology of GBM is characterized by high cellularity, pleiomorphic cells with nuclear atypia, prominent mitotic activity, microvascular proliferation, and necrosis; either ischemic or palisading [10]. Specifically, pseudopalisades are hypercellular zones that surround necrotic foci in GBM and are severely hypoxic [11]. The extent of necrosis and, especially, palisades have been negatively correlated with survival [12]. According to the fifth edition of the world health organization (WHO) Classification of Tumors in the Central Nervous System (WHO CNS5), GBM grading is now not solely based on histology, as the occurrence of CDKN2A/B homozygous deletion leads to a central nervous system (CNS) grade IV diagnosis, even if there is no microvascular proliferation or necrosis present [13].

### Molecular profiles associated with GBM

GBM contains a varied genetic profile that differs between isocitrate dehydrogenase (*IDH*)—wild type and *IDH*—mutant one [1]. The majority of primary GBM is characterized by IDH wild-type genotype, telomerase reverse transcriptase (*TERT*) mutations [4], epidermal growth factor receptor (*EGFR*) amplification [8], alterations in receptor tyrosine kinase signaling pathways in tumor protein p53 (*TP53*) [6], allelic loss of phosphatase and tensin homolog (*PTEN*) and epigenetic dysregulation, such as methylguanine methyltransferase (*MGMT*) promoter methylation [14], and loss of heterozygosity (LOH) 10q23 presentation [9] and high levels of CD44 [15] (Table 1). According to the most recent WHO classification of tumors of the CNS, the identification of *IDH* wild-type GBM relies on the detection of *EGFR* amplification, *TERT* promoter mutation, or the simultaneous occurrence of entire chromosome 7 gain and entire chromosome 10 loss (+7/−10) [16] (Table 1).

**Table 1:** Most common molecular biomarkers and signaling pathways associated with GBM

	Biomarkers		Signaling pathways
	Primary GBM	Secondary GBM	
Previous biomarkers	*IDH* wild type	*IDH* mut.	*IDH* mut.
	*EGFR* ampl.	*TP53* mut.	*EGFR*
	*TERT* mut.	*MGMT* methyl.	*TP53*
	*TP53* mut.	*ATRX* mut.	*ATRX*
	*MGMT* methyl.	1p/19q co-deletion	*PDGF*
			*BRAF* ^*V600E*^
New Biomarkers	Chromosomal (+7/−10)	LOH 10q25	*VEGF*
	*PTEN* mut.		*PTEN*
	LOH 10q23		*MET*
	+CD44		*HIF*-1α
			*PD*-1
			*NOTCH*

Note: Mut.: mutant; ampl.: amplification; methyl.: promoter methylation.

*IDH*-mutant GBM is less common [1], characterized by *TP53* [6] and alpha-thalassemia/mental retardation X-linked (*ATRX*) mutations [5], loss of chromosomes 1p and 19q [9,17], LOH of chromosome 10 [8], originating from cells in the frontal lobe, in contrast to the widespread distribution of *IDH*-wildtype, which may stem from cells in the subventricular zone [17,18] (Table 1). In contrast to the *IDH*-wild type, the *IDH*-mutant is associated with improved survival in GBM patients [19].

### Hypoxia and HIF-1α—VEGF in association with GBM

GBM is characterized by the presence of hypervascularization and necrosis, both caused by a hypoxic microenvironment [4]. Hypoxic microenvironment or “Hypoxia” is considered the pathological state in which there is inadequate oxygen supply to the tissues [20].

HIF-1 is a dimeric protein complex that regulates oxygen homeostasis within cells. Specifically, HIF-1 is a heterodimeric transcription factor consisting of a constitutively expressed β-subunit and an oxygen-regulated α-subunit. These genes contain a basic helix-loop-helix (bHLH) motif and Per-ARNT-Sim (PAS) domain, both of which aid in dimerization and the binding of the subunits to DNA [4]. Under hypoxic conditions, the HIF-1 protein is stable and active as hydroxylase and can interact with its co-activators and can dimerize with its constitutively expressed β-subunit. Once stabilized, the HIF-1 protein can bind to the regulatory regions of its target genes, inducing the expression of several genes [21,22]. HIF-1α can regulate more than 100 target genes involved in hypoxia-mediated apoptosis, angiogenesis, and cell proliferation, rendering it crucial for the histology, pathophysiology, and management of GBM [21,22]. In particular, HIF-1α induced by hypoxia increases oxygen delivery, such as by stimulating angiogenesis with the transcription of the VEGF [23]. HIF-1α has been associated with GBM tumor progression and treatment resistance, among others [22]. In the GBM tumor microenvironment, HIF expression is elevated promoting an increase of nitric oxide (NO) which may contribute to tumor growth by promoting neovascularization encompassing angiogenesis. Angiogenesis in GBM contributes to the growth and highly vascularized nature of these tumors. However, abnormal vasculature during this process further leads to hypoxia and HIF activation [4].

Angiogenesis plays a critical role in GBM aggressiveness and poor prognosis. During neovascularization, the new pathological vessels cause a low oxygen supply to the tumor, hence generating necrosis. In addition, the newly formed vessels are characterized by morphological alterations, including the formation of fenestrations and disrupted tight junctions. This implicates a disrupted Blood–Brain–Barrier (BBB), as well, leading to fluid leakage and vasogenic edema [24]. GBMs become more aggressive as they learn to adapt to this microenvironment and are characterized by drug resistance, due to low drug delivery to the tumor [21].

VEGF is an important factor associated with vasculogenesis and angiogenesis. Its main target is endothelial cells, but it also acts on other cell types [21]. The VEGF family includes VEGF-A, which is the most important factor regarding angiogenesis during homeostasis and disease, as well as VEGF-B, VEGF-C, and the placental growth factor (PGF) [25]. Not only is VEGF essential for physiologic vascular homeostasis in all body tissues, but it also plays a key role in the molecular mechanisms of tumor growth and metastasis [25].

It has been documented that VEGF plays a key role in the biology of GBM. The release of VEGF and other angiogenic factors is stimulated by the hypoxic and necrotic environment within the GBM cells. Its secretion, then, leads to the proliferation, migration, and survival of the epithelial cells via binding to the VEGF receptor (VEGFR). Both VEGF and VEGFR are highly expressed in GBMs [26]. In particular, VEGF mRNA expression was found to be increased in high-grade gliomas compared to low-grade gliomas, while its expression was high in the necrotic areas of the tumor, leading to increased angiogenesis and tumor progression. Thus, VEGF and VEGFR can be very useful as GBM prognostic biomarkers [27], while vascular-targeted drugs, such as anti-VEGF Ab, are considered an attractive therapeutic approach against vascularized GBM [28].

When a tumor has grown enough in size, usually more than 2 to 3 mm^3^, then the pre-existing circulation is not enough to meet its needs for oxygen supply [29], due to mass and/or the obstruction of the nearby blood vessels and therefore the disruption of the perfusion of the surrounding tissue [30]. Consequently, tumor cells try to adapt to this harsh environment, mostly via the HIF-1α/VEGF pathway. [31,32]. HIF-1α binds to the HRE (hypoxia-responsive elements) in the promoter region of the VEGF gene, leading to the recruitment of transcriptional factors, like p-CREB and p-STAT3, to the promoter region [33]. This cascade leads to VEGF mRNA transcription. VEGF mRNA is highly expressed after only a few hours of hypoxic state and its levels get back to normal after oxygen supply to the cells is restored.

VEGFs and VEGFR play a crucial role in angiogenesis and lymphangiogenesis, especially under hypoxia [34]. Hence, in GBM, there is an increasing body of data on HIF-1, its subunit HIF-1α, and VEGF expression regarding their relationship and possible involvement in prognosis and tumor progression.

## Methods

We explored the literature for HIF-1α and VEGF expression in GBM by performing a systematic review. We used the terms {“Glioblastoma multiforme” [OR] “Astrocytoma Grade IV” [OR] “Anaplastic Astrocytoma Grade III” [OR] “Anaplastic Astrocytoma Grade IV”} [AND] {“HIF-1α”} [AND] {“VEGF”} [AND] {“immunohistochemistry” [OR] “immunoassay” [OR] “Elisa” [OR] “western blot”} from 1998 up to April 2024 (Pubmed; Medline/Embase), following the principles of PICO [35]. Two separate teams worked on this search and reviewed all titles and abstracts. Full articles were retrieved from any article deemed relevant by either reviewer. Data were extracted from relevant methodological articles and reviewed by an independent reviewer.

We used inclusion criteria such as (i) evaluation of GBM prognosis based on HIF-1α/VEGF expression, (ii) evaluation of GBM prognosis based on other biomarkers related to HIF-1α/VEGF, (iii) evaluation of GBM prognosis based on clinical and imaging manifestations in GBM related to HIF-1α/VEGF expression, (iv) application of immunohistochemistry or other immunoassay for protein expression, as well as (v) evaluation of the effectiveness of GBM therapeutic strategies based on HIF-1α/VEGF expression. We used exclusion criteria, such as data not reporting both HIF-1α and VEGF or prognosis.

We extracted data regarding GBM prognosis and immunophenotypes of (i) HIF-1α and VEGF, (ii) HIF-1α/VEGF and IDH, (iii) HIF-1α/VEGF and other related prognostic markers, (iv) HIF-1α/VEGF and clinical and imaging manifestation in GBM. (v) Finally, we extracted data regarding HIF-1α/VEGF immunophenotypes and GBM therapeutic strategies. Methodological assays for HIF-1α/VEGF evaluation and GBM cell lines, animal or human survival assessment were examined for the extracted data.

## Results

**Figure 1:**
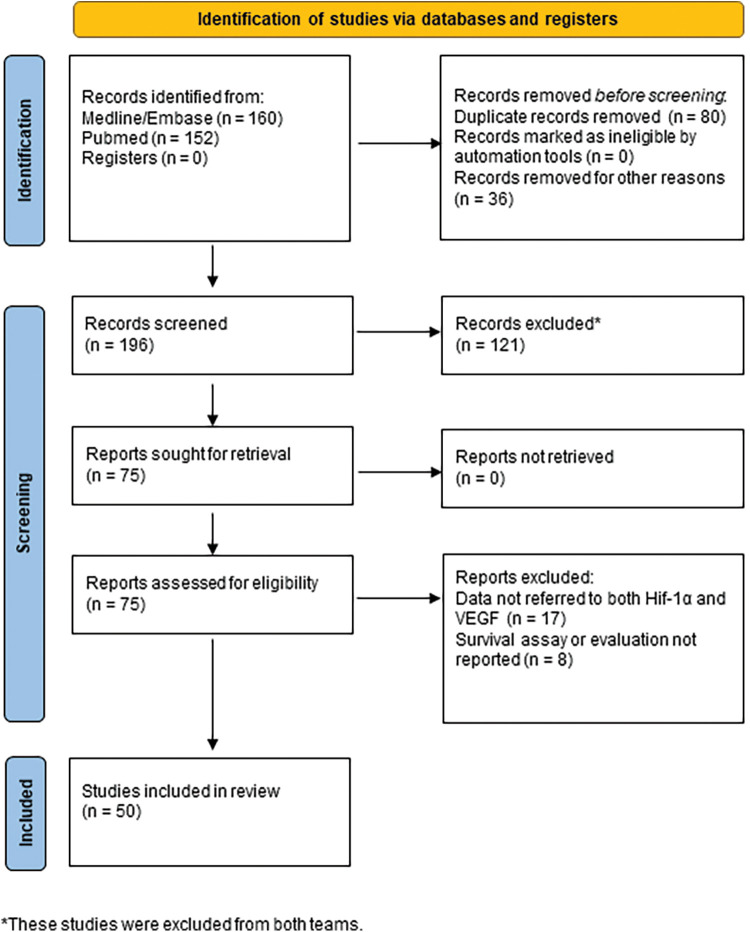
Diagram of included studies (by PRISMA 2020).

Our search revealed fifty full-text articles (Fig. 1) by PRISMA 2020 [36], including in total 1319 GBM human specimens, 18 cell lines or GBM-derived stem cells, and 6 different animal models. The main details of the studies regarding the clinicopathology of HIF-1α/VEGF in GBM are demonstrated in Tables 2–6.

**Table 2:** HIF-1α and VEGF immunophenotypes in GBM

Author (year)	*Sample size/type	Methods	Results	Survival correlation
D’Alessio et al. (2016) [40]	50 GBM	IHC	Both **HIF-1α** and **VEGF** expression are associated with **peritumoral niche** early tumorigenic events.	N/A
Hendriksen et al. (2009) [37]	E106	IHC, WB	**VEGF** expression is independent of **HIF-1α** at the **early onset** of angiogenesis.	Adverse
Korkolopoulou et al. (2004) [39]	53 GBM	IHC	**HIF-1α** and **VEGF** were associated with the GBM histological grade.	Adverse
Birner et al. (2003) [41]	114 GBM	IHC	**HIF-1α** and **VEGF** markers **distinguished** classic and bizarre **vascular formations** correlated to survival.	Adverse
Chan et al. (1998) [38]	20 GBM	ISH	**VEGF** and **VEGFR** were upregulated in all GBM, while a rather regional pattern of staining was observed in lower grades.	N/A

Note: *GBM: patients or cell line (E106); IHC: immunohistochemistry; WB: western blot analysis.

**Table 3:** HIF-1α/VEGF and IDH immunophenotypes in GBM

Author (year)	*Sample size/type	Methods	Results	Survival correlation
Potharaju et al. (2019) [47]	87 GBM	IHC	**HIF-1α nuclear** staining was found in both **wild-type and mutant IDH1**, around necrotic GBM regions.	Adverse
Chen et al. (2019) [19]	42 GBM	IHC	HIF-1α οverexpression was found in IDH-wild-type tumor sites.	Adverse
Polívka et al. (2018) [46]	52 GBM	IHC	**Lower** expression of **VEGF** was observed in GBM with **IDH1 mutation**.	Adverse
Yalaza et al. (2017) [44]	32 GBM	ELISA	**HIF-1α** and **VEGF** were found to be significantly **increased** in **IDH1-mutated** GBM.	Adverse
Zhao et al. (2009) [45]	U87MG	IHC	**HIF-1α** was **highe**r in GBM with **IDH1 mutation** relative to those with wildtype IDH1.	N/A

Note: *GBM: patients or cell line (U87MG); IHC: immunohistochemistry; ELISA: enzyme-linked immunosorbent assay.

**Table 4:** HIF-1α/VEGF and IDH immunophenotypes and related prognostic markers in GBM

Author (year)	*Sample size/type	Related biomarkers	Methods	HIF-1α/VEGF related	Survival correlation
D’Amico et al. (2023) [64]	U87MG, A172, 1 GBM	ADNP, HIF-1α, VEGF, GFAP	IHC, Immunofluorescence Analysis, Western Blot, ELISA	**ADNP may positively regulate** the expression or activity of **HIF-1α and VEGF**.	Ν/Α
Zhang et al. (2023) [65]	LN229, U251MG, 42 GBM)	CD206, CD80, HIF-1α, CD163, CDK2, CDK6, Cyclin D1, CD133, OCT4, CD68, VEGF, EGF, TGF-β1, VEGFR, Nrf, etc.	qPCR, Western Blot, ELISA	Hypoxic conditions promoted **M2 polarization** in TAMs through upregulating **HIF-1α**. Hypoxic M2 macrophages secreted **VEGF**, which activated the PI3K/Akt/Nrf2 pathway.	Adverse
Wang et al. (2020) [57]	U87MG, 15 GBM	HIF-2α, miR-210-3p	IHC, IF, ELISA, WB, qPCR	HIF-1α upregulated by **miR-210**, but HIF-2α inhibited. HIF1α and **HIF-2α** regulated each other through a negative feedback loop.	Adverse
Huang et al. (2019) [56]	LN229, BALB/c nude mice	ATG5, miR-224-3p	IHC, WB, qPCR	**HIF-1α**, **ATG5** and **VEGF** negatively regulated the expression of **miR-224-3p** under hypoxic conditions.	Adverse
Tsai et al. (2018) [59]	LN229, GBM8401, xenograft mice	PLOD3	WB, ELISA, qPCR, IHC	**PLOD3** correlated positively with grade. Knockdown of PLOD3 inhibited HIF-1α and VEGF.	Adverse
Zhang et al. (2018) [79]	U251MG, U373	STAT1	IHC, WB	**STAT1** inhibited HIF-1α and VEGF-A expression.	Positive
Chiba et al. (2017) [31]	52 GBM, KS-1	ALK	IHC, ISH, FISH, WB, qPCR	Knockdown of **ALK** downregulated **STAT3**/ HIF-1α and VEGF-A	Positive
Ishii et al. (2016) [32]	21 GBM, T98G	CA IX, SOX2, NANOG, HIF-1α, RNApII-S2P	IHC	Cells positive for HIF-1α and **CA-IX** were similarly distributed in the zones around **necroses**—distinct cell subpopulations were exclusively found in GBM tissues.	Adverse
Takahashi et al. (2015) [71]	U251MG, BALB/c nude mice	pAKT, AKT, Oct3/4	IHC, ICC, ELISA, qPCR	**Oct-3/4** overexpression promotes tumorigenic and angiogenic abilities by increased levels of VEGF meditated by the induction of **AKT**/ HIF-1α.	Adverse
Agrawal et al. (2014) [55]	U87MG, U251MG, 30 GBM	miR-210-3p	Luciferase assay, RT--PCR	**HIF-1α** regulates **miR-210-3p.** Increased miR-210-3p leads to **HIF-1α** and **VEGF** overexpression and related to CA9.	Adverse
Clara et al. (2014) [61]	208 GBM	PDGF-C	IHC	Concomitant upregulation of **PDGF-C**, **CD105** with VEGF in both GBM cells and vessels—with positive proliferative markers—indicated a correlation with hypoxia, neo-angiogenesis, and proliferative potential.	Adverse
Wang et al. (2014) [75]	U87MG	FIH-1	IHC, qPCR, WB	Overexpression of **FIH-1** resulted in the downregulation of both **GLUT-1** and **VEGF-A**, under normoxia and hypoxia.	Positive
Korkolopoulou et al. (2007) [60]	52 GBM	CA-IX,	IHC	**CA-IX** expression is associated with angiogenic markers, proliferative potential, morphology of micro-vessels, and histologic grade.	Adverse
Perdiki et al. (2007) [67]	51 GBM	COX-2	IHC	**COX-2** correlated with the angiogenic factors, the proliferative activity and total vascular area.	Adverse
Zagzag et al. (2006) [58]	18 GBM, LN308, U87MG	CXCR4	IHC, WB, qPCR	**CXCR4** correlated with HIF-1α expression and increased in hypoxia. VEGF in part upregulates CXCR4.	N/A

Note: *Patient specimens (GBM), cell lines (U87MG, A172; LN229, KS-1, T98G, U251MG, LN308), or animal model (xenograft BALB/c mice); IHC: immunohistochemistry; ELISA: enzyme-linked immunosorbent assay.

**Table 5:** HIF-1α/VEGF and clinical and imaging manifestation in GBM

Author (year)	*Sample size/type	Methods	Results	Survival correlation
Berendsen et al. (2019) [83]	76 GBM	IHC, qPCR	Decreased **HIF-1α**/**STAT5b,** as well as associated with EMT and **CEBP-β**, in **epileptogenic** tumors.	Positive
McGahan et al. (2017) [80]	43 GBM	IHC, microarray, MRI	Angiogenic expression was correlated with hemorrhagic GBM.	N/A
Bekaert et al. (2017) [82]	24 GBM	IHC, ^18^F-FMISO PET	Expression of all **hypoxia** and **angiogenesis markers** was significantly higher in the ^**18**^**F-FMISO** uptake group.	Adverse
Beppu et al. (2015) [85]	13 GBM	IHC, MRI, ^18^F-FRP170 PET	High uptake areas on ^18^F-**FRP170 PET** represented **hypoxic** l**esions** while unclear for areas with proliferative activity.	N/A
Beppu et al. (2013) [86]	12 GBM	IHC, MRI, ^18^F-FRP170 PET	High uptake areas of ^18^F-**FRP170** corresponded to **hypoxic areas**.	N/A
Belloli et al. (2013) [88]	F98, Fisher rats	IHC, MRI, ^18^F-FAZA PET, ^18^F-FDG PET	**HIF1-α** and **VEGF** immunophenotypes fitted with ^18^F-FAZA PET analysis. High **VEGF** positivity surrounded oedematose areas while **HIF1-α** was localized in hypoxic peritumoral and less in perinecrotic areas.	Adverse
Shibahara et al. (2010) [87]	3 GBM	IHC, MRI, ^18^F-FRP170 PET	High uptake areas of ^18^F-**FRP170** signified **hypoxic locations**.	N/A

Note: *Patient specimens (GBM), cell line (F98), or animal model (Fisher rats); IHC: immunohistochemistry; MRI: Magnetic Resonance Imaging; PET: positron emission tomography; ^18^F-FMISO: ^**18**^F-fluoromisonidazole; ^18^F-FRP170: 1[2-fluoro-1-(hydroxymethyl)ethoxy]methyl-2-nitroimidazole; ^18^F-FAZA: ^18^F-flouroazomycin arabinoside; ^18^F-FDG: ^18^F-fluorodeoxyglucose.

**Table 6:** HIF-1α/VEGF and therapeutic strategies in GBM

Author (year)	*Sample size/type	Treatment agent	Results
Bramatti et al. (2024) [113]	U87MG	TMZ, TmHg	**TmHg** decreased GBM cell viability and **inhibits HIF-1α**. The expression of VEGF was unchanged by either TmHg or TMZ. Co-exposure to **TmHg and TMZ** increases cytotoxicity in GBM cells and significantly **reduced HIF-1α, VEGF and p-STAT3**.
Liu et al. (2020) [92]	170 GBM, U87MG, U251MG, xenograft mice	TMZ	**Ferritin Light Chain (FTL)** was u**pregulated** in HIF-1α–dependent way, indicating resistance to TMZ therapy.
Navone et al. (2018) [104]	5 GBM	ASA	ASA combined with other treatments **decreased hypoxic, angiogenic, proliferative**, and **anti-apoptotic** factors and increased **proapoptotic** signaling.
Tamura et al. (2016) [102]	6 GBM	Neoadjuvant BEN	**Reduction** of **MVD, HIF-1α**, and **CA-IX** but varying results for VEGF and its receptors.
Khan et al. (2017) [105]	U87MG, DBTRG, patient-derived neurosphere	FLVM, FLVZ	Administration of derivatives of caffeic acid, FLVM, and FLVZ, resulted in a significant **decrease** in **HIF-1α, CD34, VEGF, IL17A, Ki67.**
Yao et al. (2017) [107]	U87MG, U251MG, C57BL6J mice	LBH589 (HDAC inhibitor)s	The histone deacetylase inhibitor Panobinostat (LBH589), displayed **antiangiogenic effects**, though HIF-1α degradation.
Gillespie et al. (2015) [115]	U87-LucNeo	siRNA for HIF-1α	HIF-1α transcriptional targets and cell growth markers were significantly lower.
Fan et al. (2014) [119]	F98, C6, GL261 U87MG, U251MG, T98G, primary rat and human brain astrocytes	DEXA	Administration of corticosteroid inhibits growth of glioma cells and tumor-induced angiogenesis due to **VEGF downregulation**.
Chen et al. (2014) [118]	U87MG, LN229, U251MG	mR-23b sponge	**Decreased** expression of **markers** of **hypoxia, migration**, and mixed about invasion.
Chandrasekaran et al. (2013) [94]	U251MG, Nude/athymic mice	RT	After RT, ^18^F-FLT uptake was reduced, and proliferative activity was attenuated. Angiogenic markers were increased, due to repairing mechanisms.
Towner et al. (2013) [110]	F98, U87MG, xenograft rats	OKN-007	**Decreased hypoxia** and **cell proliferation**, **increased apoptosis**, mixed for angiogenesis, and not significant difference in cell differentiation.
Wei et al. (2011) [117]	4 gCSCs	WP1066 (inhibitor of STAT3)	Inhibition of STA3 downregulates HIF-1α, VEGF and hypoxic-induced immunosuppressive effects.
Méndez et al. (2010) [114]	LN308, U87MG, GL261, C57BL6J mice	shRNA for HIF-1α	Knockdown of HIF-1α **counteracted** its **aggressive** and **invasive potential** both *in vitro* and *in vivo*.
Fisher et al. (2007) [8]	U251MG, U87MG	TMZ	TMZ **activated stress mechanisms** in GBM cells that included the angiogenesis-inducing proteins **HIF-1α and VEGF**.
Kamiyama et al. (2005) [103]	U87MG, U251MG,	SN38 (active metabolite of IRI)	**Decreased HIF-1α** and **VEGF** expression of glioma cells in a dose- and time-dependent manner under normoxic and hypoxic conditions.
Lund et al. (2004) [93]	U87MG	IR	**HIF-1α** was **upregulated,** as well as **VEGF** even under normoxia. Angioproteins and Glut-1 were not affected.
Lin et al. (2016) [108]	U87MG, HA-h, BALB/c *nu*/*nu* mice	CRLX101 (nanoparticle)	Decreased levels of CA-IX, VEGF and CD31.
Reardon et al. (2009) [101]	27 GBM	Etoposide and BEN	**Lower CA-IX** but **increased VEGF** expression was associated with better survival among GBM patients

Note: * Patient specimens (GBM), cell lines (U87MG, U87-LucNeo; U251MG, LN229, LN308, GL261, DBTRG; F98, C6, T98G, primary rat, and human astrocytes; patient-derived neurosphere; HA-h; gCSCs or GBM associated cancer stem cells), or animal models (murine xenografts; Nude/athymic mice; C57Bl6J mice; rat xenografts; BALB/c *nu*/*nu* mice). FLVM: di-amine caffeate/rosmarinate; FLVZ: imidazole caffeate/rosmarinate; RT: Radiotherapy; TMZ: Temozolomide; TmHg: thimerosal: ASA: acetylsalicylic acid or Aspirin; BEN: Bevacizumab; DEXA: dexamethasone; IRI: irinotecan; IR: irradiation; SN38: active metabolite of irinotecan.

### HIF-1α and VEGF immunophenotypes in GBM

GBM is the most malignant of all astrocytomas and with the poorest prognosis, too. The main histopathologic features that contribute to its high malignancy are the pseudopalisades around necrotic areas, due to hypoxia.

We found five studies investigating the expression of HIF-1α and VEGF in GBM from 237 patients and one cell line (Table 2), through IHC. It was shown that malignant cells in GBM present HIF-1α nuclear and/or VEGF cytoplasmic immunoreactivity [37–39] (Table 2).

**Figure 2:**
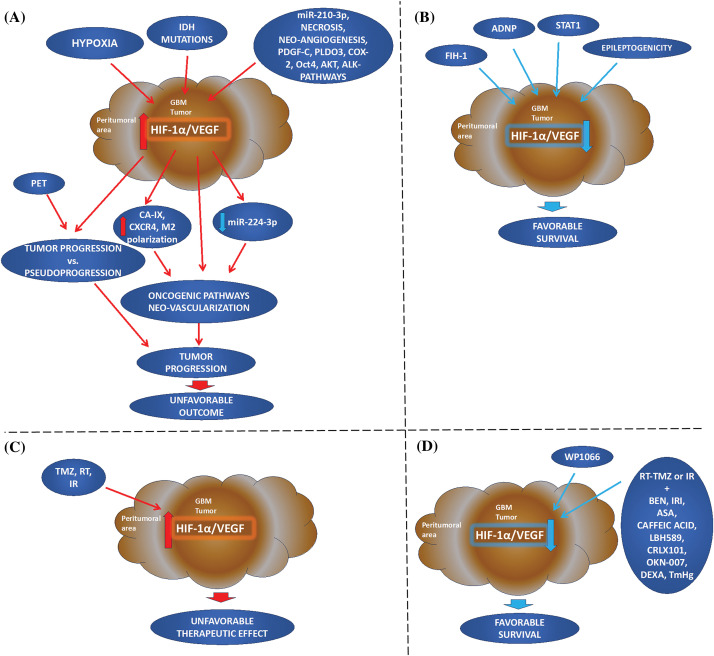
Hypoxia-inducible factor 1alpha and vascular endothelial growth factor in Glioblastoma Multiforme: going beyond pathologic implications. (A, B) Cellular mechanism: Elevated HIF-1α and VEGF immunophenotypes in the tumor or peritumoral areas are induced by hypoxia and may related to *IDH* tumor mutations and other oncogenic factors leading to deregulation of other factors, neo-vascularization, and tumor progression. HIF-1α/VEGF immunostaining can distinguish PET macroscopic data of pseudoprogression from tumor progression. On the other hand, FIH-1, and STAT1 appear to be critical in downregulated HIF-mediated genes, important for angiogenesis, or/and hypoxic-induced immunosuppression. Epileptogenicity also appears to have a more favorable survival of patients, its molecular profile has been characterized by down-regulation of HIF-1α/hypoxia. (C, D) Treatment: First-line treatment (TMZ, RT, or IR) can result in increased HIF-1α and VEGF expression with an unfavorable prognosis. However, the combination of first-line treatment with other therapeutic strategies can reduce HIF-1α and VEGF resulting in a favorable tumor prognosis and patient survival. Also, blocking STAT3 by WP1066, inhibits hypoxic-induced tumor growth factors, HIF-1α and VEGF, and immunosuppression (IDH: isocitrate dehydrogenase; PDGF-C: platelet-derived growth factor C; COX-2: cyclooxygenase-2 inhibitor; PLDO3: procollagen-lysine, 2-oxoglutarate 5-dioxygenase; Oct4: octamer-binding transcription factor 4; ADNP: activity-dependent neuroprotective protein; AKT: AKT serine/threonine kinase or protein kinase B; CXCR4: C-X-C chemokine receptor type 4; CA-IX: carbonic anhydrase; M2 polarization; FIH-1: factor inhibiting HIF-1; WP1066: inhibitor of JAK2 and STAT3; STAT1: signal transduction and activator of transcription 1; PET: positron emission tomography; RT: radiation therapy; TMZ: temozolomide; IR: irradiation; BEN: bevacizumab; IRI: irinotecan; ASA: acetylsalicylic acid or Aspirin; LBH589: histone deacetylase inhibitor Panobinostat; CRLX101: cyclodextrin-based polymer (CDP) and camptothecin-containing nanoparticle drug conjugate; OKN-007: Oklahoma nitrone-007; DEXA: dexamethasone; TmHg: thimerosal).

HIF-1α, VEGF, and VEGFR expression were identified in both GBM and peritumoral tissue, but HIF-1α and VEGF expression increased in cells within the tumor, whereas VEGFR density was low in both tumoral and peritumoral tissue cells [40] (Table 2, Fig. 2). However, neo-angiogenesis is present in the GBM-neighboring areas, even under normoxic conditions, and is exclusively due to VEGF [37] (Table 2). The vascular patterns that occur in GBM present both classic and bizarre angiogenic sub-types, with the more classic the pattern being distributed, the longer the survival [41] (Table 2).

### HIF-1α/VEGF and IDH immunophenotypes in GBM

*IDH1* mutations comprise the core of the molecular identity of GBM and serve as the main feature for distinguishing between histopathologically similar cases. *IDH* mutations—either IDH1 or IDH2—result in the production of the oncometabolite 2-hydroxyglutarate (2-HG), which in turn promotes the oncogenic properties of hypoxia and vascularization [42]. *IDH1/2* mutations enhance the expression of HIF-1α—dependent proteins [43].

We found five studies, including 213 GBM and one cell line, explored HIF1a and/or VEGF and IDH by IHC analyses or ELISA (Table 3). *IDH1* mutations were found to increase HIF-1α and VEGF serum levels in primary GBM and contribute to oncogenesis through the induction of the HIF-1α pathway in primary GBM, even under normoxia, a process named “pseudohypoxia” [44,45] (Table 3, Fig. 2). However, increased levels of HIF-1α have been also found in GBM patients with wild-type *IDH1*, relative to normal tissue [19,46,47] (Table 3); leading to an inconsistency necessary to be clarified.

### HIF-1α/VEGF immunophenotype in GBM prognosis

GBM incident is diagnosed usually in older people (average of 55 years) [48], particularly in individuals with a wild type IDH (mean of 62 years), while it can occur in younger age (mean of 44 years) with a mutated IDH [10]. Age constitutes a risk factor due to the time-dependent accumulation of cellular damage that occurs. However, the expression of HIF-1α has not been associated with age [47] (Table 3).

### HIF-1α/VEGF immunophenotypes and other factors of hypoxia, angiogenesis, proliferation, and metabolism in GBM prognosis

The overexpression of hypoxia-related markers is a pivotal element of the GBM phenotype. We found fifteen studies, including 490 GBM, 9 different cell lines, and BALB/c nude or xenograft mice investigating the association of HIF-1α/VEGF expression with other factors, in GBM prognosis. Specifically, HIF-1α/VEGF immunophenotypes were found to be associated with the expression of other prognostic factors, related to hypoxia, including deregulated miRNAs, tumor *necrosis*, neo-angiogenesis, Carbonic anhydrase (CA-IX), cyclooxygenase-2 inhibitor (COX-2), octamer-binding transcription factor 4 (Oct4), AKT serine/threonine kinase or protein kinase B (AKT), and C-X-C chemokine receptor type 4 (CXCR4), tumor-associated macrophages (TAMs), activity-dependent neuroprotective protein (ADNP), as well as with hypoxia-independent factors, such as ALK (Anaplastic lymphoma kinase) (Table 4, Fig. 2).

MiRNAs are small non-coding RNAs consisting of ~20–24 nucleotides, that control gene expression in ~30% of human genes. They recognize and bind to the 3′ untranslated region of mRNAs, therefore increasing or decreasing its target production [49]. The deregulation of miRNAs has been documented during carcinogenesis [50–52], which can be prevented or reversed by using specific inhibitors of related pathways [53,54]. It has been also reported that an association between HIF-1α and miRNA deregulation in GBM. Specifically, HIF-1α can regulate miRNAs, through binding in the promoter regions of the hypoxia-related miRNAs (HRM) and *vice versa*. This bidirectional regulation is of high importance in the regulation of tumor progression, considering that HIF-1α is involved in tumor angiogenesis through a variety of mechanisms including a regulatory mechanism of miRNAs [49,55].

Based on the literature, the expression of a panel of miRNAs, such as miR-210-3p, miR-224-3p, miR-1275, miR-376c-3p, miR-23b-3p, miR-193a-3p, miR-145-5p, 92b-3p, miR-20a-5p, miR-10b-5p, miR-181a-2-3p and miR-185-5p, is deregulated by hypoxia [55,56]. Predominant miRNAs such as miR-224-3p and miR-210-3p have been examined in GBM concerning hypoxic markers, HIF-1α and HIF-2α, and other markers [56,57] (Table 4).

Specifically, high levels of miR-210-3p were found to be accompanied by high levels of VEGF and CA9, while the transcription of VEGF and CA9 was found to be mediated by HIF-1α, implying high correlation of miRNAs to the hypoxia within the GBM tumor [55] (Table 4). Also, under hypoxia, it was shown a negative correlation in GBM between miR-224-3p and HIF-1α, VEGF, or ATG5 (autophagy-related gene 5) [56], a key molecule of autophagy, while downregulation of miR-224-3p led to an increase cell mobility and chemoresistance [56] (Table 4).

Several studies have confirmed that both HIF-1α and VEGF are strongly expressed in GBM, and both present a remarkably similar distribution around areas of necrosis that correlate with tumor grade and are associated with poor prognosis [58–60] (Table 4). These areas were also found positive for PLOD3 and CXCR4, previously associated with modification of collagen and the extracellular matrix (ECM) and epithelial to mesenchymal transition (EMT) [58,59] (Table 4).

Although HIF-1α positivity is a common finding in GBM cases, there are noticeable areas where cells are either HIF-1α positive or negative. In the HIF-1α positive areas, the extent of vascularity is increased as determined by micro-vessel density (MVD) measurements [60,61] (Table 4). In these areas, the neoangiogenic and proliferative influence of VEGF was confirmed since cells positive for VEGF were also found positive for CD105, a membrane glycoprotein known to distinguish normal vessels from malignant neovascularization [62], as well as for PDGF-C (platelet-derived growth factor C) expression [61] (Table 4). The participation of PDGF-C in angiogenesis has already been demonstrated and it can be postulated that in areas of hypoxia, PDGF-C may indirectly induce angiogenic activity via upregulation of VEGF or even directly by activation of PDGF-Ra, -Rb receptors [63].

Carbonic anhydrase (CA-IX) and VEGF are both products of hypoxia-induced pathways and are known as downstream regulated targets of HIF-1α. Specifically, HIF-1α, VEGF, and CA-IX exhibited a remarkably similar distribution in GBM cases [32] (Table 4); however, HIF-1α and VEGF immunoreactivity levels were higher compared to CA-IX levels and were represented with a more diffuse pattern [60] (Table 4). Both CA-IX and VEGF expression were found to be significantly correlated with WHO tumor grade in astrocytic gliomas, however, CA-IX and VEGF positivity did not correlate with each other [62]. Moreover, four different mechanisms have been related to the upregulation of VEGF in gliomas, only one of which relates VEGF to CA-IX [62].

ADNP, an intracellular astrocyte-derived neurotrophic factor that is essential for brain development, was recently found to be expressed in hypoxic areas of GBM modulating the hypoxic-angiogenic pathway by reducing VEGF secretion, acting as a tumor suppressor [64].

M2 phenotypes of TAMs are cancer-associated lymphocytes that have been related to GBM’s poor prognosis and hypoxic conditions through secreting VEGF [65].

COX-2 is an isozyme that is rapidly induced under pathological conditions, often associated with inflammatory processes. It has been implicated in the progression of a variety of tumors; most brain tumors showed constitutively elevated levels of COX-2 and among them GBM tumors, where COX-2 was upregulated mostly in central, hypoxic regions of the tumor [66]. COX-2 expression was found to be positively correlated with VEGF and HIF-1α expression, as well as total vascular area in GBM cases [67] (Table 4). Prostaglandin E2 (PGE2), the predominant product of COX-2 activity, has been shown to cause increased VEGF expression, indicating that COX-2-mediated angiogenic effect could be attributed to PGE2 activity [68]. Hence, VEGF expression in gliomas could be regulated both through HIF-1α and COX-2 pathways [67].

Oct4, a well-known regulator of differentiation in embryonic stem cells, was also expressed in human gliomas and over-expressed in high-grade gliomas. Therefore, the malignancy in gliomas could be related to the presence of stem-like cells in the tumor. Oct4 is expressed in rat C6 glioma cells and neural stem cells [69]. On the other hand, Oct4 is induced by HIF-2α, while both HIF-2α and HIF-1α are required for the induction of VEGF expression in glioma stem cells [70]. It has been suggested and could be postulated that in GBM cells, hypoxia-induced-HIF-2α upregulation of Oct4, which in turn has been shown to induce AKT, can activate HIF-1α, thus leading to VEGF activation and angiogenesis [71] (Table 4).

The proto-oncogene AKT has also been shown to modulate HIF-1α and VEGF protein expression through the PI3K/PTEN/AKT/FRAP pathway in cancer cells [72]. In GBM cells, AKT signaling stabilizes HIF-1α, while the deregulation of AKT activity through loss of the tumor suppressor protein PTEN during malignant progression contributes to tumor expansion [73]. Similarly, analysis of GBM biopsy samples showed that loss of PTEN was highly correlated with activation of AKT, which in turn was correlated with phosphorylation of downstream effector molecule mTOR [74]. However, PTEN is not as potent as the factor inhibiting HIF-1 (FIH–1), especially in hypoxia, as it has been suggested that FIH-1 appears to be more critical than the loss of PTEN in HIF activation in GBM cells under hypoxia. FIH-1 overexpression leads to transcription inhibition of the HIF-mediated genes, important for angiogenesis, such as GLUT-1 and VEGF-A, thus contributing to chemosensitivity [75] (Table 4).

CXCR4 expression, a G-protein coupled receptor involved in the epithelial-to-mesenchymal transition (EMT) and cancer stem cell survival, was correlated, in GBM tumors, with a state of progression and therapy resistance [76]. Additionally, in GBM patients, CXCR4 increased levels were associated with poor prognosis [77]. Since CXCR4 levels were elevated in tumor and vascular cells of GBM, it was suggested that pseudopalisade cells around hypoxic areas of necrosis overexpress CXCR4 under the control of HIF-1α. In addition, CXCR4 upregulation in endothelial cells could be attributed to VEGF released by the pseudopalisading cells [58] (Table 4).

Anaplastic lymphoma kinase (ALK) is associated with the tumorigenesis of human cancers, including GBM tumors [78]. It has been proposed that N-myc and Sox4-dependent ALK overexpression activates downstream transduction cascades involving increased STAT3, AKT, HIF-1α, and VEGF-A expression, resulting in increased cell proliferation and neovascularization [31] (Table 4).

STAT1, a prototypical member of signal transducer and activator of the transcription (STAT) protein family, is downregulated under hypoxic conditions in GBM cells, while its overexpression can inhibit HIF-1α and VEGF-A expression, as well as decrease proliferation, migration, and invasion of GBM cells [79] (Table 4).

### Clinical and imaging manifestation of GBM in the context of HIF-1α/VEGF

We found seven studies explored in 171 GBM and 1 animal model of the association of HIF-1α/VEGF immunophenotypes with clinical and macroscopic image analysis data for GBM prognosis.

Hemorrhage and epileptogenicity have been assessed as the main symptomatology of GBM that derives from HIF-1 and *VEGF* [80]. Hemorrhage, which is present in many GBM cases, has been linked to increased expression of angiogenic markers (CD34 and CD105), of some angiogenic genes—(HIF-1α and MDK) which share similar mechanisms of induction [81], and decreased expression of the Coagulation factor III (F3), enhancing susceptibility to hemorrhage. However, hemorrhage has not been associated with prognosis [80]. On the other hand, epileptogenicity appears to favor the survival of GBM patients [82]. Specifically, the biomarker profile of epileptic GBM patients compared to non-epileptic cases has been characterized by down-regulation of HIF-1α/hypoxia gene sets and STAT5b target genes, as well as reduced nuclear-phosphorylated STAT5b protein expression [83] (Table 5, Fig. 2). In addition, in epileptic GBM patients, gene sets involved in epithelial-to-mesenchymal transition (EMT) and CEBP-β signaling were found to be down-regulated, however, the expression of the key mesenchymal transcription factors NF-κB p65, STAT3, and CEBP-β was not altered [83] (Table 5). Activation of EMT signaling is indicative of GBM progression potential, hence, epileptogenicity seems to favor the survival of GBM patients [84].

In addition, various studies have associated the degree of hypoxia through the macroscopic image [via PET (positron emission tomography) hypoxia tracers and MRI (Magnetic Resonance Imaging)] and hypoxic and angiogenic markers immunophenotypes (by IHC) [82,85–87] (Table 5). Correlation of MRI—PET imaging and HIF-1α or VEGF immunophenotype has been reported, possibly allowing monitoring of tumor progression *in vivo* [88] (Table 5). Although imaging techniques demonstrate satisfying results in the representation of hypoxic lesions, they have not managed, yet, to distinguish between tumor progression and pseudoprogression, a process that occurs post-treatment and presents lesions similar to tumor progression [89]. Therefore, it is necessary to combine macroscopic evaluation with histopathological features.

### Therapeutic strategies and challenges based on HIF/VEGF

The treatment of GBM has been challenging and often not radical, leading to recurrences and eventually shortened survival. The mechanisms behind treatment failure have been partly elucidated, with hypoxia playing the most crucial role by promoting both chemo- and radioresistance [6,90,91], as well as cell heterogeneity, resulting in various subpopulations [32]. We found eighteen studies investigated in 208 GBM, 15 cell lines or GBM-derived stem cell cultures, and 5 animal models, several therapeutic approaches concerning HIF-1α/VEGF (Table 6, Fig. 2).

Temozolomide (TMZ), combined with radiotherapy (RT) consists of a first-line treatment, found to significantly prolong survival in GBMa patients. It has been reported, however, that TMZ activates stress mechanisms that can induce HIF-1α expression, which in turn upregulates VEGF, and thus may result in an unfavorable therapeutic effect [8] (Table 6). In this regard, novel biomarkers regarding prognosis, as well as response to TMZ therapy in glioma have been proposed [92] (Table 6). Similarly, RT has been found to upregulate VEGF, however, by mechanisms independent of HIF-1 transactivation [93] (Table 6). Other studies have shown that irradiation (IR) can upregulate HIF-1α/VEGF, modestly compared to hypoxia-induced expression, signifying a secondary silent tumor repair activity, despite its successful treatment, as indicated by the decreased 18F-FLT PET uptake and the increased γH2AX [94] (Table 6).

The above data suggests that first-line treatment should be supplemented with either antibodies against VEGF (e.g., Bevacizumab) or antibodies against other angiogenic factors induced by hypoxia to improve clinical results.

Bevacizumab (BEN) has been the main part of the treatment of recurrent GBM due to its anti-VEGF effect. Despite the promising preclinical data [95,96] anti-VEGF monotherapy, including BEN, has failed to improve patients’ overall survival [97–100]. A prior study reported that under neoadjuvant BEN there was a significant reduction of micro-vessel density, as well as HIF-1α and CA-IX expressions [101], however, the results on VEGF and its receptors were mixed [102] (Table 6).

Irinotecan (IRI) often supplements BEN as it has been shown that HIF-1α and VEGF expression are decreased in a dose- and time-dependent manner under normoxic and hypoxic conditions [103] (Table 6). Aspirin (acetylsalicylic acid or ASA) has been also examined against GBM. ASA appears to act synergistically with TMZ or BEN, as it decreased the expression of the hypoxic (HIF-1α, VEGF, VEGFR1/2), proliferative (HRAS, MEK, ERK, PI3K, AKT) and the anti-apoptotic signaling pathways (BCL-2; B-cell lymphoma 2), while the pro-apoptotic signaling (BAX; Bcl-2-associated X protein) was increased [104] (Table 6).

Derivatives of caffeic acid (FLVM and FLVZ) inhibited IL17A and its mediated growth factor VEGF [105] (Table 6). IL17A has been shown to promote angiogenesis via direct upregulation of VEGF [106].

LBH589, a histone deacetylase inhibitor Panobinostat, displayed significant antitumor effects on GBM such as inducing HIF-1α instability, degradation, and decreased VEGF expression [107] (Table 6).

CRLX101, an investigational cyclodextrin-based polymer (CDP) and a camptothecin-containing nanoparticle drug conjugate, inhibited both hypoxia and angiogenesis by decreasing the levels of VEGF, CD31, and CA–IX [108] (Table 6). This therapeutic approach has been studied in other types of cancers, too, and demonstrated increased effectiveness by affecting HIF-1α [109].

OKN-007 (Oklahoma nitrone-007), a novel anti-glioma nitrone-based agent, resulted in a significant decrease in hypoxia (HIF-1α) and cell proliferation (MIB-1), an increase of apoptosis (cleaved caspase 3), while showed mixed results about angiogenesis, such as decreased micro-vessel density (MVD) but not of VEGF, and no effect on cell differentiation (CA-IX) [110] (Table 6). In a recent study, the evidence about angiogenesis became more clarified as OKN-007 combined with TMZ, resulted in a significant decrease in tumor progression by targeting the tumorigenic TGF-β1 pathway, which promotes angiogenesis among others [111].

Thimerosal (TmHg), a known inhibitor of the thioredoxin system with a history of clinical use, appears as a promising therapeutic strategy to increase ROS levels and oxidative stress and induce GBM cell apoptosis. Ethylmercury (EtHg) derived from the metabolism of TmHg is particularly effective in inhibiting the thioredoxin system [112]. A recent investigation showed that exposure to TmHg or EtHg decreased GBM cell viability in a time-dependent manner [113] (Table 6). It was shown that exposure of GBM cells to TmHg led to an overall decrease in the nuclear accumulation of HIF-1α. This study also showed that the expression of VEGF was unchanged by either TmHg or TMZ. However, co-exposure of GBM cells to TmHg and TMZ reduced tumor growth-related factors, HIF-1α and VEGF, and p-STAT3 [113] (Table 6).

Small interfering RNA (siRNA) against HIF-1α has also been utilized by several studies. Knockdown of HIF-1α reduced migration *in vitro* and invasion *in vivo* as well as the ability of murine glioma cells to form tumor spheres [114] (Table 6). IHC analysis, in mice receiving daily HIF-1α siRNA injections has confirmed the macroscopic image of reduction of tumor volume through the decreased levels of HIF-1α transcriptional targets, like VEGF, GLUT–1, c-MET, and CA-IX and markers for cell growth like MIB-1 and MVD [115] (Table 6).

STAT3 is a signal transduction and activator of transcription factor, which has been shown to play a role in GBM development and progression [116]. Inhibition of STAT3 can downregulate HIF-1 and VEGF and inhibit tumor growth and angiogenesis. Specifically, targeting JAK and STAT3 with WP1066 in GBM-associated stem cells can effectively lead to downregulation of HIF-1α and VEGF expression and reverse the hypoxic-induced immunosuppression [117] (Table 6).

miRNA sponge for miR-23b, which contains multiple target sites that are complementary to miR-23b, has been also used to diminish the malignant phenotype of GBM. MiR-23b has a tumor suppressor function in GBM. Application of miRNA sponge for miR-23b in GBM was found to reduce tumor malignancy, through the downregulation of HIF-1α, VEGF, and other molecules, suggesting miR-23 as a promising anticancer therapy either alone or in combination with current targeted therapies [118] (Table 6).

Dexamethasone (DEXA) has been largely utilized as an adjuvant treatment modality, preferably in the early stages of GBM diagnosis, to make tumor-microenvironment more prone to therapy and lengthen survival time. An *in vitro* study has associated corticosteroid administration with dampened cell growth due to the downregulation of VEGF and reduction in abnormal vascular formation, and vasogenic edema formation, and vasogenic edema [119] (Table 6).

## Discussion

We performed a literature review to evaluate the potential use of HIF-1α/VEGF immunophenotype alone, as well as with other prognostic factors for GBM prognosis and therapeutic approaches. Our research revealed that HIF-1α nuclear and/or VEGF cytoplasmic immunoreactivity is strongly associated with malignant cells in GBM [37–41]. Specifically, increased immunopositivity of HIF-1α and VEGF is associated with early tumorigenic events [40]. Notably, VEGF may be independently activated at the early onset of angiogenesis [37], while increased HIF-1α/VEGF immunoreactivity is associated with GBM progression [39]. Based on our data, increased expression of HIF-1α and VEGF is associated either with wild-type or mutant *IDH* [19,44–47]. *IDH—*mutation is related to oncogenic properties of hypoxia and vascularization [42,43], however, *IDH—*mutations can also induce HIF-1α oncogenic pathway even under “pseudohypoxia” [44,45]. These data lead to an inconsistency that needs to be clarified by further HIF-1α/VEGF and IDH analyses by IHC in GBM. However, HIF-1α*/*VEGF immunoreactivity has been associated with other prognostic factors of GBM, including hypoxia-related deregulated miRNAs, necrosis, neo-angiogenesis, CA-IX, COX-2, Oct4, AKT, ADNP, M2 polarization, and CXCR4, as well as with hypoxia-independent prognostic factors, such as ALK related pathways (Fig. 2).

Based on our data, HIF-1α may be involved in GBM tumor angiogenesis through a regulatory mechanism of miRNAs, such as miR-210-3p and miR-224-3p [49,55–57]. These miRNAs may interact either with the hypoxia-mediated pathways, to promote or inhibit angiogenesis [55,57], or with autophagy factors, to increase cell mobility and chemoresistance [56].

HIF-1α and VEGF immunophenotypes show a remarkably similar distribution around areas of tumor necrosis, which is associated with GBM tumor grade and poor prognosis [58–60]. These areas were also found positive for PLOD3 and CXCR4, supporting an unfavorable prognosis [58,59] (Fig. 2). However, there are noticeable areas of GBM where HIF-1α positive cells present extent malignant neovascularization, and increased VEGF positivity, accompanied by increased positivity for CD105 [62], a malignant neovascularization marker, or PDGF-C [61], which stimulates angiogenesis and revascularizes ischemic tissue. These data support a direct or indirect-induced angiogenic activity, via the activation of PDGF-Ra, -Rb receptors [61] or PDGF-C, promoting VEGF upregulation [62], respectively.

Both HIF-1α and VEGF exhibited a remarkably similar distribution with CA-IX in GBM [32], which is regulated by HIF-1α, similar to VEGF, and correlated with tumor grade [62]. In addition, increased HIF-1α and VEGF immunoreactivity are positively correlated with COX-2 [7], which may be particularly elevated in hypoxic areas of GBM [64,65], while COX-2 angiogenic effect has been attributed to PGE2 activity [66]. This data suggests that VEGF expression in gliomas may be regulated by both HIF-1α and COX-2.

Other hypoxic-induced prognostic factors, such as Oct4, AKT, ADNP, M2 polarization, and CXCR4 are associated with HIF-1α/VEGF [64,65,71,73,74]. Specifically, Oct4 can activate HIF-1α, leading to angiogenesis through VEGF activation [71], while AKT can modulate HIF-1α and VEGF protein expression through the PI3K/PTEN/AKT/FRAP during GBM development [73,74]. Similarly, hypoxia induced M2 polarization can activate the oncogenic PI3K/Akt/Nrf2 pathway through the secretion of VEGF [65]. Also, CXCR4 is overexpressed in pseudopalisading cells of hypoxic necrotic areas in GBM. This observation suggests that HIF-1α-induced activation of CXR4 is attributed to VEGF released by the pseudopalisading cells [58]. In addition, activation of hypoxia-independent pathways related to ALK can promote STAT3, AKT, HIF-1α, and VEGF-A expression, resulting in increased cell proliferation and neovascularization [31], contributing to GBM tumorigenesis [78]. On the other hand, FIH-1, ADNP, and STAT1 appear to be critical in the activation of HIF and angiogenesis-related genes, under hypoxia [64,75,79], acting as tumor suppressors in GBM (Fig. 2).

HIF-1α/VEGF immunophenotypes have also been associated with clinical and Imaging manifestations of GBM (Fig. 2). These may include hemorrhage and epileptogenicity [80]. Hemorrhage has not been associated with prognosis [80]. However, epileptogenicity is one of the main symptomatology of GBM, which appears to favor the survival of GBM patients [81]. Epileptogenicity has been characterized by down-regulation of HIF-1α/hypoxia gene sets and STAT5b target genes, and CEBP-β and EMT signaling-associated gene sets, the latter is indicative of GBM progression potential [81]. Also, *in vivo* and pilot clinical studies have reported HIF-1α or VEGF immunophenotypes fitted with the correlation of PET-imaging [82] and/or MRI [85–88], supporting the combined macroscopic evaluation with histopathological features in monitoring [88] and distinguishing GBM tumor progression from pseudoprogression [89] (Fig. 2).

Finally, several GBM therapeutic approaches have been considered in the context of HIF-1α/VEGF immunophenotypes, due to hypoxia-induced chemo- and radioresistance in GBM (Fig. 2). TMZ, RT or IR can activate HIF-1α and VEGF, with unfavorable results [8,92–94], suggesting that first-line treatment should be supplemented with either antibodies against VEGF (e.g., BEN) or antibodies against other angiogenic factors induced by hypoxia, to improve clinical results. These therapeutic approaches may include TMZ combined with BEN [102], IRI [103], ASA [104], caffeic acid [105], LBH589 [107], CRLX101 [108], OKN-007 [110], TmHg [113], siRNA or shRNA against HIF-1α [114,115], and miRNA sponge [118], as well as corticosteroids [119]. This combination can decrease tumor progression by reducing the expression of hypoxic and angiogenic factors, including HIF-1α and VEGF. Neoadjuvant BEN can reduce micro-vessel density, as well as HIF-1α and CA-IX expressions [102], with mixed results for VEGF [88], but when supplemented with IRI can reduce both HIF-1α and VEGF [103]. ASA can also act synergistically to TMZ or BEN, suppressing the hypoxic (HIF-1α, VEGF, VEGFR1/2) and oncogenic or antiapoptotic pathways [104]. Other drugs, such as LBH589 or CRLX101 also exhibit antitumor effects by reducing HIF-1α and VEGF expression [107,108], while OKN-007, a novel anti-glioma nitrone-based agent, can also be synergically to TMZ inducing a significant decrease in tumor progression by targeting TGF-β1 pathway-promoting angiogenesis [111]. Derivatives of caffeic acid can also suppress VEGF through IL17A in GBM [105]. Recent investigation supports that thimerosal (TmHg) alone or in combination with existing chemotherapeutic drugs, such as TMZ, can reduce GBM cell viability, and cellular response to hypoxia, as well as neoangiogenesis and should be considered to moderate GBM progression [113].

Notably, siRNA against HIF-1α can reduce the ability of murine glioma cells to migrate [114] accompanied by decreased immunophenotype levels of HIF-1α transcriptional targets, including among others VEGF, and CA-IX [118]. In addition, blocking JAK/STAT3 can modulate Hif-1α and VEGF upregulation in GBM cells and inhibit hypoxic-induced immunosuppressive effect, suggesting STAT3 is an effective target for GBM [117]. In addition, miRNA sponge for miR-23b can reduce GBM tumor malignancy, through the downregulation of HIF-1α, VEGF, and other molecules, suggesting miR-23 as a promising anticancer therapy either alone or in combination with current targeted therapies [118].

## Conclusion

GBM is a complex entity, with a clinicopathological behavior that delineates its resilience to therapy. The GBM pathologic pattern varies within the same specimen, implying the involvement of several oncogenic pathways. Hypoxia is a main coordinator behind the complex molecular cascades, via the expression of HIF-1α and the activation of its numerous gene-targets. Angiogenesis emerges by HIF-1α activation, through the protein family of VEGF. HIF-1α/VEGF immunophenotypes correlate with other prognostic factors, and oncogenic signaling pathways, such as JAK/STAT3 and PI3K/AKT. Reduced HIF-1α/VEGF immunophenotypes also correlate with FIH-1, ADNP, or STAT1 upregulation, as well as with clinical manifestations, like epileptogenicity, and a favorable prognosis of GBM. In parallel, data from both MRI–PET imaging and HIF-1α or VEGF immunophenotypes can distinguish between GBM tumor progression and pseudoprogression. Finally, HIF-1α/VEGF immunophenotypes can reflect GBM treatment efficacy, including combined first-line treatment with histone deacetylase inhibitors, thimerosal, or an active metabolite of irinotecan, as well as STAT3 inhibitors alone, and resulting in a favorable tumor prognosis and patient survival. Our data support HIF-1α/VEGF’s role as biomarkers of GBM prognosis and treatment efficacy. Further insights into the HIF-1α and VEGF immunophenotypes could also document their use as biomarkers in GBM treatment efficacy, including ongoing clinical trials [91,100,120,121].

The hypoxic microenvironment is the main feature that confers GBM in its aggressiveness and treatment resistance [4,20,21]. We showed that new strategies are tested to overcome the GBM-associated hypoxic-induced activation of tumor growth factors, including HIF-1α and VEGF, and immunosuppression, with promising results. Due to the central role of immunotherapy in the investigation of cancer treatment, further investigation including research strategies of modulation of the immune tumor microenvironment, such as the GBM-associated and hypoxia-induced HIF-1α/VEGF pathways would contribute to the treatment of GBM. Therefore, gaining more evidence of the role of HIF-1α/VEGF and related signaling pathways in GBM progression will further support the use of their immunophenotypes in prognosis and the effectiveness of the treatment of GBM.

## Data Availability

All data generated or analyzed during this study are included in this published article and are available from the corresponding author upon reasonable request.

## Abbreviations

GBM: Glioblastoma multiforme
HIF-1α: Hypoxia-Inducible Factor 1alpha
VEGF: Vascular Endothelial Growth Factor
